# Body weight of 35-day-old broilers is associated with proximal small intestinal inflammatory and oxidative pathways – A multi-omics study

**DOI:** 10.1016/j.psj.2026.106463

**Published:** 2026-01-18

**Authors:** Samuel C.G. Jansseune, Wouter H. Hendriks

**Affiliations:** a Animal Nutrition Group, Department of Animal Sciences, Wageningen University & Research, Wageningen, the Netherlands; b Université Paris‐Saclay, INRAE, AgroParisTech, GABI, Jouy‐en‐Josas, France; c Idena, Sautron, France

**Keywords:** Broiler, Body weight, Pathway analysis, Inflammation, Oxidative stress

## Abstract

A major growth performance trait of modern-day broiler chickens is the ability to attain a high body weight (**BW**) within a short time. Here we identified parameters or factors significant associated with higher broiler BW using data on tissue (blood, jejunum, ileum and caecal tonsil) transcriptome, ileal and caecal digesta microbiota and metabolome, and blood biochemical and immune parameters. The data originated from 35-day-old Ross 308 male broilers reared under practical housing conditions and fed a non-starch polysaccharide-rich diet supplemented with either a probiotic or a postbiotic, including or not their carriers. Omics data were available for 72 broilers which were a subset of the 160 birds for which blood biochemical and immune parameters were available. The distribution of the BW did not significantly deviate from normality within the treatment groups and overall. Among all datasets, the jejunal tissue transcriptome was most associated with differences in broiler BW. Notably, Kyoto Encyclopedia of Genes and Genomes (**KEGG**) pathways associated with inflammatory response were significantly (*p* < 0.05) enriched in broilers with a low BW (e.g. Toll- and NOD-like signaling, phagosome, cytokine-cytokine receptor interaction, and cytosolic DNA sensing pathways) while gene ontology (**GO**) pathways associated with a response to toxic substances and anti-oxidative defenses were enriched in birds with high BW. In caecal tonsil tissue, broilers with a higher BW had pathways related to the immune response enriched (KEGG: cytokine-cytokine receptor interaction; GO: immune system process and response) with minor changes observed in their microbiota. Broiler BW was mainly associated with the tissue transcriptome, especially jejunum. In broilers with a higher BW, decreased expression of inflammation and increased expression anti-oxidative defense pathways were observed in jejunal tissue, while the caecal tonsil tissue showed a higher expression of immune pathways. Reducing inflammation and increasing anti-oxidative defenses in the proximal small intestine of broilers may provide future targets for improved broiler growth.

## Introduction

Poultry production has consistently increased over the past decades and is nowadays the number one meat consumed worldwide (OECD/FAO, 2021). Poultry meat production is expected to further increase to 153 million tons by 2030, representing 41% of the global meat production (OECD/FAO, 2021). The world poultry meat production in 2022 was dominated by chicken [89.6%] followed by turkey [4.6%] and duck [3.8%] (Petracci, 2022). Broiler chicken production efficiency has increased significantly over the past decade, with a reduction in feed conversion ratio of approximately 1% per year (de Haas et al., 2021). Besides meat quality, this major trait of growth performance by broilers to reach a high body weight (**BW**) with a low feed intake, is one of their main advantages over other terrestrial production animals.

The gut is a major organ involved in broiler growth performance that ensures selective nutrient supply for growth while at the same time, protecting the host from being invaded by pathogens (Wickramasuriya et al., 2022). The small intestine is the major site of nutrient digestion and absorption, while the caeca are a major site of microbial fermentation. Both the small intestine and the caeca have an innate and adaptive immune system with the caecal tonsils being particularly relevant when studying immune-related processes in caeca (Haghighi et al., 2008; Setta et al., 2012; Cazals et al., 2022). The gut microbiota impact the health of the host, interact with immune cells, and can modulate gut morphological structure in broilers (Diaz Carrasco et al., 2019; Liao et al., 2020; Fathima et al., 2022; Kogut, 2022). Studies have reported associations between the gut microbiota, mainly in the caeca, and growth performance in broilers (Han et al., 2016; Lee et al., 2017; Lundberg et al., 2021), but a recent standardized literature survey concluded that there is limited consistency in the results of correlational studies of bacterial taxa with growth performance, health and nutritional status (Marková et al., 2024). A less often studied intermediate between the microbiota and the host is the metabolome. Its composition results from dynamic processes including metabolite production and metabolization by microbial and host cells. The most studied microbial metabolites are the short-chain fatty acids (Liu et al., 2021) although they provide a limited view of the overall metabolome. In contrast, (un)targeted metabolomics allows for detailed determination of a large number of metabolites (Gertsman and Barshop, 2018), and as such, constitutes a more powerful analytical tool to investigate the gut metabolome in broilers.

In addition to the intestine, the blood also fulfils an important role in growth by transporting nutrients and waste as well as transporting signaling molecules between organs. The blood is a dynamic tissue that reflects the physiological status of the animal (Hanash et al., 2011). In that regard, blood transcriptome analyses were concluded to be informative of the immune and health status (Chaussabel, 2015). Although, the blood is relatively easily accessible through a minimally invasive sampling procedure and, as such a good tissue for marker identification, only a few studies have investigated the chicken’s blood transcriptome, particularly when compared to other farm animal species of higher economic interest such as cows and pigs (Chaussabel, 2015).

Over recent years, omics analyses including metagenomic, metabolomics and transcriptomics have increased in popularity as they allow the large-scale screening for association with a parameter of interest. In the study of Jansseune et al. (2025a; b), the blood, jejunal, ileal and caecal tonsil tissue transcriptome, ileal and caecal digesta microbiota (16S metagenomic) and metabolome (volatile fatty acids and semi-polar untargeted metabolome) were measured, as well as a multitude of blood biochemical and immune parameters in 72 broilers at 35 days-of-age. The latter broilers were all reared under practical housing conditions which differs from experimental facilities e.g. in term of air quality and sanitary pressure or stocking densities. For example, it has been reported that the intestinal and caecal digesta microbiota composition of broilers and laying hens differed greatly depending on housing facilities (Stanley et al., 2013; Kers et al., 2019; Marcolla et al., 2023; Pires et al., 2024). The study presented by Jansseune et al. (2025a; b) was designed to investigate effects of multiple dietary conditions, but also additional biological questions when proper statistical tools are employed. The dataset can, for example, be used to investigate how BW is associated with differences in the tissue metabolism, gut digesta microbiota and metabolome.

The present study was conducted to investigate associations and interactions between BW on the one hand and the blood, jejunal, ileal and caecal tonsil tissue transcriptome, ileal and caecal digesta microbiota and metabolome, and blood biochemical and immune parameters on the other hand. The aim was to identify, using the available datasets, parameters or factors that are associated with BW in broilers.

## Materials and methods

The data used for the current study originated from a single study, the data of which were published in two articles (Jansseune et al., 2025a; b). A summary of this experiment is presented below.

### Ethic statement

The experiment was approved by the French Ministry of Education, Higher Education and Research (Ministère de l'Éducation nationale, de l'Enseignement supérieur et de la Recherche) under the protocol No. APAFIS #44135-2023071114126771 v5, and carried out according to the French legislation. The birds were euthanized by electronarcosis followed by cervical dislocation.

### Birds housing and management

A large batch of one-day-old male Ross 308 broilers was purchased from a commercial hatchery (Couvoir de Cleden, Cleden Poher, France), with 1,600 chicks selected, based on individual weights and distributed across 40 pens with 40 broilers each, so that all pens had a similar average chick body weight (**BW**) (∼44.9 ± 2.42 g) and distribution. Pen size was 1.90 × 1.25 × 0.8 m (*L* × *W* × *H*) with wood shavings as floor covering. Birds were located along the wall of air entries on one side of a commercial, 1200 m² Colorado-type building. Water and feed per pen were provided *ad libitum*. A pelleted diet was formulated based on commercial standards for nutrient levels for Ross 308 broilers and provided adequate levels of all nutrients to the birds (Table 1). The diet (**Ctrl**) was supplemented with a probiotic or a postbiotic purchased from STI biotechnologie (Maen Roch, France) with or without a carrier (Jansseune et al., 2025a; b). Each dietary treatment had 8 pen replicates. As in normal practice, at the day of hatch, broilers were spray vaccinated against infectious bronchitis virus (**IBV**) (Nobilis BI H120, Nobilis, MSD santé animale, Beaucousé, France) and coccidiosis (Paracox-5, Intervet UK Ltd, United-Kingdom), and were vaccinated through drinking water against infectious bursal disease virus (**IBDV**) (HIPRAGUMBORO G97, Laboratorios Hipra, Amer, Spain) on d17. At d14, an induced immune reaction using vaccines for Newcastle disease virus (**NDV**) and IBV (Nobilis Ma5 + CLONE 30, MSD Santé Animale, Beaucouzé, France) booster shot was administered via an individual eye drop.

**Table 1:** Ingredient and calculated composition including energy content of the standard and challenge starter (0-14 d), grower (14-28 d) and finisher (28-37 d) diet for broilers.

Composition	Starter	Grower	Finisher
**Ingredient (% as is)**			
Corn	13.982	12.509	13.000
Wheat	28.000	30.000	29.949
Barley	10.000	10.000	10.000
Rye	7.500	12.500	18.000
Soybean meal	32.000	26.800	20.700
Limestone	1.640	1.110	0.850
Mono calcium phosphate dihydrate	1.400	0.860	0.800
Sodium chloride 99%	0.280	0.250	0.260
Sodium bicarbonate	0.100	0.110	0.130
Soy oil	3.890	4.650	4.890
DL-methionine 99%	0.301	0.274	0.231
L-Lysine HCL 98%	0.242	0.257	0.329
L-Threonine 98%	0.134	0.129	0.163
L-Valine 96.5%	0.031	0.024	0.051
L-Isoleucine 90%	-	0.027	0.067
L-Arginine 98%	-	-	0.080
Premix^1^	0.500	0.500	0.500
**Calculated (% as is)**			
Dry matter	88.8	88.9	88.8
Crude protein	21.5	19.6	17.5
Crude fat	5.28	6.02	6.28
Starch	36.9	40.0	43.3
Ash	6.61	5.26	4.65
Fibre^2^	13.9	13.7	13.3
Total non-starch polysaccharides^2^	12.4	12.2	11.8
Soluble non-starch polysaccharides^2^	3.05	2.98	2.85
Cellulose	2.86	2.76	2.63
Dig. Methionine	0.59	0.54	0.47
Dig. Methionine+cystine	0.93	0.86	0.75
Dig. Lysine	1.32	1.20	1.10
Dig. Threonine	1.32	1.20	1.10
Dig. Valine	0.90	0.82	0.75
Dig. Arginine	1.29	1.14	1.05
Calcium	0.98	0.67	0.55
Available phosphorous	0.48	0.35	0.33
Chlorine total	0.27	0.25	0.27
Sodium total	0.15	0.14	0.15
Apparent metabolizable energy (MJ/kg as is)	12.13	12.72	12.88

1 Supplied per kg premix: 2,000,000 IU retinyl acetate, 500,000 IU cholecalciferol, 10 g DL-α-tocopherol, 460 mg menadione, 400 mg thiamine, 1,500 mg riboflavin, 700 mg pyridoxine-HCL, 4 mg cyanocobalamin, 7 g niacin, 2.4 g d-pantothenic acid, 92 g choline chloride, 200 mg folic acid, 40 mg biotin, 53 g FeSO_4_·H_2_O, 9.6 g CuSO_4_·5H_2_O, 28 g MnO, 33 g ZnSO_4_·H_2_O, 360 mg KI, 112 mg Na_2_SeO_3_.

2 Values were calculated from levels reported by Knudsen (2014) and restricted to cereals and soybean meal.

### Data used for analysis

The omics data reported by Jansseune et al. (2025a) (microbiota composition and metabolome in ileal and caecal digesta, and blood, jejunal, ileal and caecal tonsil tissue transcriptome) were obtained on a subset of three dietary treatment groups (control, probiotic and postbiotic-supplemented without carrier). The omics data were obtained from 3 broilers randomly sampled per pen replicates (*n* = 24 per treatment). The blood biochemical and immune parameters (cell count, immunoglobulins (**Ig**)A, IgM and IgY, and antibody titers against NDV, IBV and IBDV) data were reported by Jansseune et al. (2025b). These data were available from all five-treatment groups (control, probiotic and postbiotic-supplemented with or without carrier) with 4 broilers randomly sampled per pen replicate (*n* = 32 per treatment). The 72 broilers for which omics data were available were a subset of the 160 birds for which blood biochemical and immune parameters were measured.

### Statistical analyses

All data analyses were performed with R version 4.0.3 (R Core Team, 2023). Probability or adjusted probability values < 0.05 and 0.05 ≤ *p* < 0.10 were considered significant and a trend, respectively.

The effect of the dietary treatment (control, pro- or postbiotic with or without carrier) on BW was assessed by ANOVA, with inclusion of a random block factor. Variance homogeneity across treatment groups was assessed by the Levene’s test. The normality of the distribution of the BW data was assessed by the Shapiro test. The skewness and kurtosis of the BW data distribution was also calculated and their deviation from the normal distribution tested with the Jarque-Bera normality test. The effect of BW on blood biochemical and immune parameters, and α-diversity indexes was investigated as presented by Jansseune et al. (2025b) and Jansseune et al. (2025a), respectively with the modification that BW was included as the explanatory variable and the treatment as a random factor.

The effect of BW on the semi-polar metabolome was assessed as presented by Jansseune et al. (2025a), with the modifications that BW was included as the explanatory variable and the dietary treatment as a random factor. Statistics were performed only on metabolites unambiguously named (Level 1 and 2a as presented by Jansseune et al. (2025a). Then, the predictability of BW from the semi-polar metabolome and microbiota datasets per segment were investigated with a partial least square (**PLS**) analysis using the untransformed data. Briefly, the PLS model was built using the pls function [package mixOmics v.6.24.0 (Rohart et al., 2017)] with default parameters, six components, and scaling. The PLS model goodness of prediction was assessed with the perf function based on a 10-fold validation and 20 repeats and using the Q2 criterion where Q2 > 0.0975 indicates that an added dimension is beneficial to improve accuracy of the model (Lê Cao and Welham, 2021).

The effect of BW on the transcriptome per tissue and digesta microbiota per segment was analyzed as presented by Jansseune et al. (2025a) with the only modification that BW was additionally included in the models and was the studied explanatory variable. Analyzed parameters included differentially expressed genes and differentially abundant operational taxonomic units (**OTUs**) as well as gene set enrichment analysis of Gene Ontology (**GO**) and Kyoto Encyclopedia of Genes and Genomes (**KEGG**). Association between BW and β-diversity indexes was investigated with the function adonis2 (package vegan v.2.6.8) using default parameters and 10,000 permutations.

## Results

### Broiler body weight, blood biochemical and immune parameters

**Fig. 1:**
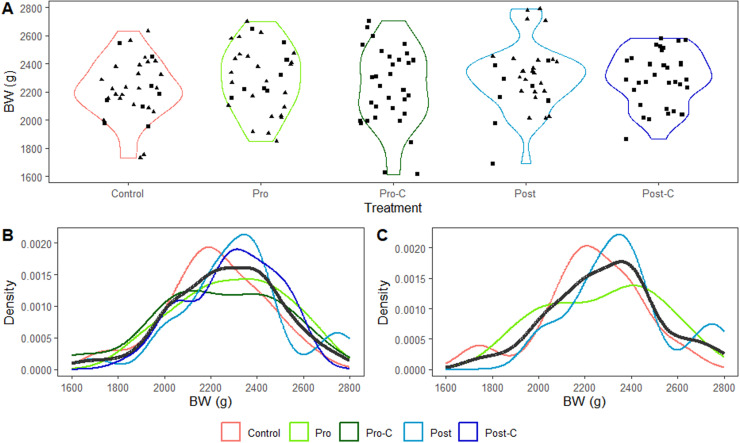
Body weight (BW) and density distribution of BW for 35-day-old male Ross 308 broilers (**A**) receiving a Control diet supplemented with a *Lactobacilli*-base probiotic (Pro) alone or with its carrier (Pro-C), or a postbiotic (Post) alone or with its carrier (Post-C). Square: Blood biochemical and immune parameters data available; Triangle: Blood biochemical and immune parameters as well as omics data available. Density distribution per treatment and overall (black line) of the (**B**) 160 broilers for which blood biochemical and immune parameter data and (**C**) 72 of the 160 broilers for which also omics data were available.

The BW distribution of the broilers included in the current study are presented per dietary treatment in Fig. 1A. The BW of the full set of 160 broilers for which blood plasma biochemical and immune parameters data were available averaged 2,269 g (min: 1,615, max: 2,108, 1st quartile: 2,108 and 3rd quartile: 2,424 g). The density plots (Fig. 1B**)** represents the distribution of the BW data points across treatments and showed that the data were normally distributed (*p* = 0.714), with no effect of the treatments on the means (*p* = 0.398) and an homogeneity of variance across groups (*p* = 0.246). The skewness and kurtosis of the overall BW distribution were −0.263 and 3.029, respectively, and the Jarque-Bera normality test, which consider both parameters, was not significant (*p* = 0.396). Per treatment group, the skewness were −0.283, −0.132, −0.365, 0.001 and −0.352, and the kurtosis were 3.035, 2.129, 2.657, 3.574 and 2.246 with the Jarque-Bera normality test not significant (*p* = 0.807, 0.576, 0.649, 0.803, and 0.492, respectively) for the control, probiotic, probiotic + carrier, postbiotic, and postbiotic + carrier groups.

Omics data were available for 72 (control, probiotic and postbiotic groups) of the 160 broilers who combined had an average BW of 2,287 g (min: 1,730, max: 2,788, 1st quartile: 2,153 and 3rd quartile: 2,425 g). The BW data of these 72 broilers had a homogenous repartition between treatment groups (Fig. 1A). The density plot (Fig. 1C) showed that the latter BW were normally distributed (*p* = 0.362) with no effect of the treatments on the means (*p* = 0.154) and an homogeneity of variance across groups (*p* = 0.382). The skewness and kurtosis of the overall BW distribution was −0.043 and 2.897, respectively, and did not differ from a normal distribution (*p* = 0.973), according to the Jarque-Bera normality test. Per treatment group, the skewness was −0.512, −0.116, and 0.466, and the kurtosis 3.404, 1.914, and 2.727, respectively, with the Jarque-Bera normality test being not significant (*p* > 0.54), for the control, probiotic and postbiotic groups.

Broiler BW was not associated (*p* > 0.1) with any of the blood plasma biochemical parameters (bile and uric acids, phosphorous, total protein, cholesterol, and triglycerides concentrations, triglyceride to cholesterol ratio, and optic density at 450 and 470 nm). Broiler BW also was not associated (*p* > 0.1) with blood plasma IgA, IgM and IgY concentration, or with antibody titers and seropositivity against NDV IBDV and IBV.

Broiler BW was negatively associated to total cells, monocytes, and cytotoxic (CD4- CD8α+) T cells counts (*p* = 0.003, 0.021 and 0.023, respectively), and tended to be negatively associated with the erythrocytes, thrombocytes, total T cells and double positive (CD4+ CD8α+) T cells counts (*p* = 0.063, 0.073, 0.094 and 0.056, respectively). For the other cell types (leukocytes, granulocytes, lymphocytes, B cells, blast B cells γδ T cells, γδ T CD8α+ cells, helper (CD4+ CD8α-) T cells and double negative (CD4- CD8α-) immature T cells counts) the effect of BW was not statistically significant (*p* > 0.1). Broilers with a higher BW tended to have a higher CD4/CD8 (*p* = 0.085), with the heterophil to lymphocytes ratio being not significant (*p* > 0.1).

### Microbiota composition in ileal and caecal digesta

Ileal and caecal microbiota α-diversity indexes (Observed, Chao1, Ace, Shannon, Simpson, inverse Simpson and Fisher) showed no significant association with broiler BW (Supplementary Figure 1A). Observed, Chao1, Ace and Fisher α-diversity indexes in the ileal digesta showed a tendency (*p* = 0.056, 0.091, 0.069 and 0.056, respectively) for a negative association with BW in a non-linear dependent manner (Supplementary Figure 1A and B). The ileal digesta microbiota β-diversity indexes (Bray, Jaccard, Unifrac and Weighed Unifrac) were not associated with BW (*p* > 0.1). However, in caeca, these β-diversity indexes had p-values of 0.036, 0.041, 0.063 and 0.186, respectively for an association with BW. A multidimensional scaling plot of the β-diversity indexes with broiler BW are presented in Supplementary Figure 2.

**Fig. 2:**
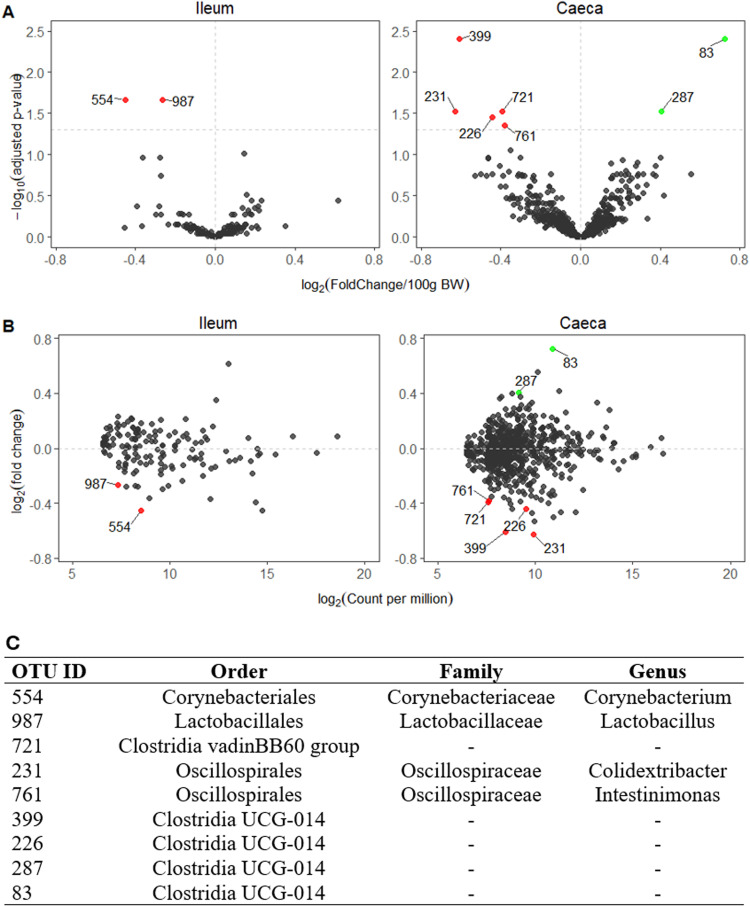
Ileal and caecal digesta microbiota operational taxonomic units (OTUs) associated with body weight (BW) in 35-day-old male Ross 308 broilers fed a non-starch polysaccharide-rich diet supplemented or not with either a probiotic or its derived postbiotic. (**A**) Volcano plot showing the differentially abundant OTUs, (**B**) their associated count per million and (**C**) phylogenetic classification of significant OTUs. In A and B, numbers represent OTU ID. Green, red and grey dots represent significantly enriched, significantly depleted and not differing OTUs, respectively. In A, the horizontal dashed line represents adjusted-*p* = 0.05.

**Fig. 3:**
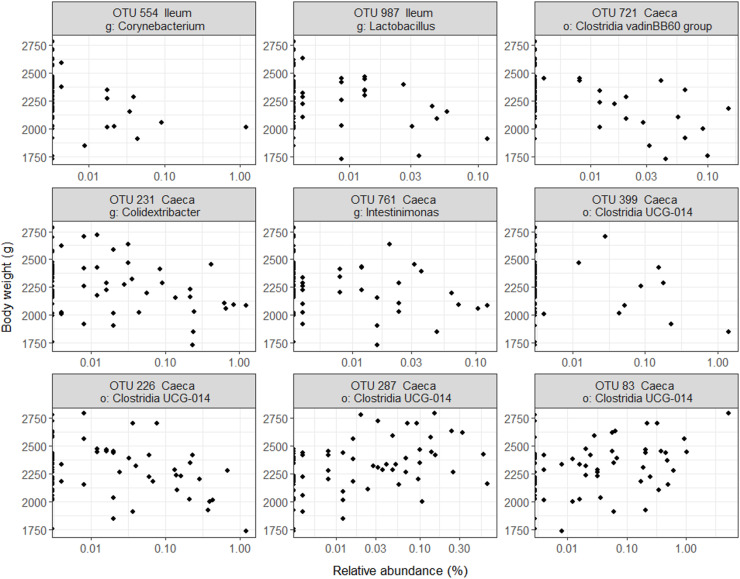
Relative abundance operational taxonomic units (OTU) in ileal and caecal digesta significantly associated with body weight in 35-day-old male Ross 308 broilers fed a non-starch polysaccharide-rich diet supplemented or not with either a probiotic or its derived postbiotic. The most detailed taxonomic identification level is presented: o: order, g: genus.

Higher broiler BW was found to be associated with two and five OTUs of lower relative abundance in ileal and caecal digesta, respectively, and two OTUs of higher relative abundance in caecal digesta (Fig. 2A). The average abundance of the latter differentially abundant OTUs ranged from 160 to 1,869 counts per million (Fig. 2B). None of the OTUs was identified at the species level but included OTUs of the genus *Lactobacillus, Corynebacterium, Colidextribacter, Intestinimonas* (Fig. 2C). The relative abundance of each of the latter OTUs is presented in Fig. 3. Four of the seven OTUs with differential abundance in the caecal digesta were of the order *Clostridia* UCG-014, out of which two increased and two decreased. The ileal and caecal digesta microbiota were not predictive of BW (Supplementary Figure 3).

### Metabolome in ileal and caecal digesta

Broiler BW was not associated with ileal and caecal digesta concentration of acetate, propionate, (iso-) butyrate, (iso-)valerate and (iso-)caproate (*p* > 0.1) (data not shown). Regarding the semi-polar metabolome, in both segments, broiler BW was not associated with different concentrations of any metabolite (adjusted-*p* > 0.1 for all) (data not shown). The ileal and caecal digesta semi-polar metabolome was not predictive of BW (Supplementary Figure 4).

### Transcriptome in blood, jejunum, ileal and caecal tissues

**Fig. 4:**
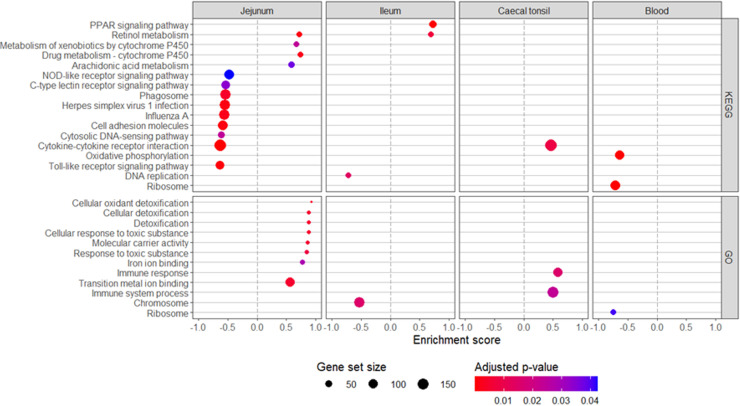
Effect of body weight on gene set enrichment of Kyoto Encyclopedia of Genes and Genomes (KEGG) and Gene Ontology (GO) pathways in blood, jejunal, ileal and caecal tonsil tissue transcriptome of 35-day-old Ross 308 male broilers fed a non-starch polysaccharide-rich diet supplemented or not with either a probiotic or its derived postbiotic.

Analyses revealed only one significant differentially expressed gene among all tissues (data not shown). Higher BW was associated with a decrease (log_2_(fold change) = −0.006/100 g BW and log_2_(count per million) = 3.76) of the protein kinase cGMP-dependent type II (*PRKG2*; Ensembl ID: ENSGALG00010003198) in the blood. Further gene set enrichment analysis revealed KEGG and GO pathways associated with lower (enrichment score < 0) or higher (enrichment score > 0) BW (Fig. 4). The cytokine-cytokine receptor interaction pathway was the only one showing an opposite significant enrichment score, which was observed between the jejunum and caecal tonsil. The retinol metabolism pathway was the only one significantly enriched with a higher BW in both jejunal and ileal tissue. Most pathways with a significant enrichment score were observed in the jejunal tissue. Notably, KEGG pathways associated with an inflammatory response were enriched in broilers with a lower BW (e.g. Toll- and NOD-like signaling, phagosome, cytokine-cytokine receptor interaction, and cytosolic DNA sensing pathways) while for birds with a higher BW, the GO pathways associated with a response to toxic substances and anti-oxidative defenses were enriched, concomitantly with enrichment of the cytochrome P450 KEGG pathways (Fig. 4). In the ileal tissue, broilers with a lower BW had enriched pathways associated with cell multiplication (GO: Chromosome, KEGG: DNA replication). In the caecal tonsil tissue, broilers with a higher BW had enriched pathways related to an immune response (KEGG: cytokine-cytokine receptor interaction; GO: Immune system process and immune response). In the blood, the ribosome (GO and KEGG) and oxidative phosphorylation pathways (KEGG) were enriched in broilers with a lower BW (Fig. 4).

## Discussion

The microbiota, certain metabolites and expression of certain genes were reported to be associated with broiler growth when studied under experimental conditions (Han et al., 2016; Lee et al., 2017; Lundberg et al., 2021; Akram et al., 2024a; b). Compared to the latter studies, here, a relatively large number of broilers (*n* = 160 for blood immune and biochemical parameters and 72 for omics data) reared under practical housing conditions were used to identify parameters and pathways potentially important to reach higher BW in context of production. The diet was high in NSP and without enzymes for experimental purposes in order to challenge broiler growth (Jansseune et al., 2024a; b, 2025a; b). The distribution of BW of the 160 and subset 72 broilers for which data were available were not significantly deviating from normality. The use of a continuous distribution of BW in the present study, rather than predetermined groups (*e.g.* Lee et al., 2017; Lundberg et al., 2021; Akram et al., 2024b), can be considered superior as it is more representative and less influenced by cut-off criteria. Of note, the BW result from 35 days of growth while the other measured parameters are continuously changing and are not only the result of what happened the 35 days before. The transcriptome, mainly in the jejunal tissue, was the variable showing the greatest associations with broiler BW, while the other variables (blood biochemical parameter, ileum and caecal digesta microbiota and metabolome) were not or in a minor way associated with broiler BW.

The jejunal tissue transcriptome interpretation through pathway analysis indicates that for broilers to reach a higher BW, reduced inflammation could play a significant role. This observation agrees with Akram et al. (2024b), who reported in a gene expression study that compared to high BW, low BW broilers had upregulated mRNA expression of pro-inflammatory immune response genes in association with increased intestinal permeability in ileal tissue. Broilers are continuously exposed to multiple factors that can promote inflammation in the intestine, such as pathogens, certain feed ingredients, high energy diets or (heat) stress (Kogut et al., 2018). Particularly, diets rich in NSP, as in the current study, were reported to promote intestinal inflammation in broilers (Chen et al., 2015; van Krimpen et al., 2017; Cardoso Dal Pont et al., 2021) and to impair broiler growth performance parameters (Jansseune et al., 2024a; b). Reduction of intestinal inflammation was proposed to be the potential primary mechanism through which antibiotic growth promoters were improving growth (Niewold, 2007; 2014). The negative association between the concentration of some blood immune cells and BW indicates a more systemic reduction of inflammation. Pro-inflammatory cytokines are known to lower feed intake, muscle deposition, and growth in broilers (Klasing, 1994; Liu et al., 2023). Furthermore, challenges which induce inflammation were reported to reduce feed intake (e.g. immune and heat stress challenge) in broilers (Remus et al., 2014; Liu et al., 2015; 2020), even if other mechanisms than inflammation are also involved.

The observed lower inflammation in the jejunal tissue of broilers with a higher BW can result from different, potentially complementary mechanisms, including higher oral tolerance to pro-inflammatory components or lower response to pro-inflammatory stimuli. Oral tolerance is a form of peripheral tolerance which explains the absence of an immune response to feed-derived proteinaceous components present in the gut which cross the epithelial barrier (Klipper et al., 2001). Interestingly, fast-growing broilers compared to layers, have a reduced production of pro-inflammatory cytokines when exposed to an immune challenge (Leshchinsky and Klasing, 2001). Broilers have also been reported to develop a more Ig mediated immunity compared to layers (Simon et al., 2014). These aspects have not been reported for broilers with low or high growth of a single breed, but it could be hypothesized that a lower inflammatory response and a more Ig oriented immunity could support the association between immunity and higher broiler BW as observed in the present study. The involvement of a more Ig mediated immunity in heavier broilers in the current experiment appears less likely as no association between blood Ig (A, M, Y) and BW was observed, in addition to the trend observed that the intestinal pathway for IgA production was enriched in jejunal tissue of lighter rather than heavier broilers (enrichment score = −0.67, adjusted-*p* = 0.062). To confirm this, dedicated studies investigating intestinal Ig production are required. The absence of an effect in the ileum as was observed for the jejunum may result from a reported lower sensitivity to inflammation by ileal tissue. Cardoso Dal Pont et al. (2023) reported that the jejunum was more responsive than the ileum in an inflammation model using high dietary NSP in broilers. The intestinal health is link to its microbiota composition (Ducatelle et al., 2023), but unfortunately, the microbiota in jejunum digesta could not be analyzed because bacterial DNA could not be extracted from the digesta in sufficient amounts although three different methods were used. In light of the results of the jejunal tissue transcriptome, these data would have been potentially explanatory for the effects on BW.

An important driver/stimulator of inflammation is oxidative stress (Biswas, 2016). In the jejunal tissue, an increase of the anti-oxidative mechanisms is suggested by the enrichment of pathways associated with anti-oxidative defenses. Whether this led to a reduction in oxidative damages remains to be investigated as pathways associated with reactive oxygen species production were also enriched. In heavier broilers, a greater need to inactivate oxidative components or metabolites is indicated by upregulation of cytochrome P450 pathways. Cytochrome P450 enzymes are involved in multiple synthesis pathways including sterols, fatty acids, eicosanoids and vitamins, and many P450s generate reactive oxygen compounds (Veith and Moorthy, 2018). The P450 enzymes are also involved in arachidonic acid (Tallima and El Ridi, 2018) and retinol metabolism (Ross and Zolfaghari, 2011). The enrichment of the retinol and arachidonic acid pathways in heavier broilers may have contributed to intestinal health. Retinol has been shown to protect lipopolysaccharide-induced damage of intestinal epithelial IPEC-J2 porcine-derived cells *in vitro* (He et al., 2019), promoted intestinal regeneration after fasting in broilers (Wang et al., 2024), and stimulated cell proliferation and enterocyte differentiation in a chicken intestinal organoid model (Wang et al., 2024). Arachidonic acid was reported to be important for paracellular permeability (Martín-Venegas et al., 2006), thereby, potentially supporting nutrient absorption.

In the caecal tonsils, three pathways associated with inflammation were enriched in higher BW broilers, indicative of a higher immune activity in this tissue. This difference between the caecal tonsil and jejunal tissue may result from their different roles. The primary role of the jejunum is the digestion and absorption of nutrients (El Sabry and Yalcin, 2023), while the caecal tonsils consist of lymphatic tissue particularly involved in immune-related processes (Haghighi et al., 2008; Setta et al., 2012; Cazals et al., 2022). The caecal lumen content is the location of the highest microbial concentration and fermentation (Ducatelle et al., 2023). A greater immune protection in this organ may contribute to protect the host from an adverse development of some micro-organism detrimental to the host. Accordingly, in the caecal digesta, OTUs potentially associated with negative effects on gut health, showed a different relative abundance depending on BW (Fig. 3). Higher *Clostridia* UGC 014 has been reported to be associated with impaired gut health in humans (Leibovitzh et al., 2022), but was also reported to be potentially associated with lower gut inflammation (Yang et al., 2021). The results in the present study regarding some *Clostridia* UGC 014 OTUs enriched in lighter or heavier broilers suggest that their association with growth and inflammation may be species dependent. *Colidextribacter* was identified as a possible predictive marker for the development of Crohn’s disease (Garay et al., 2023) and to be associated with impaired gut health in humans (Leibovitzh et al., 2022), suggesting that their decrease in relative abundance in heavier broiler may have been beneficial. However, in the absence of a complete identification at the species level, further association between differentially abundant OTUs and broiler BW would be speculative. Despite that the role of the microbiota remains largely unclear, and as it is overall not associated with broiler BW in the present study, some of the caecal microbiota β-diversity indexes were associated (Bray and Jaccard) or tended (UniFrac) to be associated with differences in BW. The β-diversity indexes are calculated differently (Kers and Saccenti, 2022), and by the way they are calculated, it is evident that the difference in β-diversity with BW was mainly due to the presence/absence of some OTUs, and with difference in the phylogeny of OTUs with low abundance.

In the blood of heavier broilers, the ribosome and oxidative phosphorylation pathways were enriched suggesting a higher cell activity in that tissue. This would agree with the higher metabolic activity required to sustain a higher growth. For a more specific, in-dept investigation into potential interaction effects within the omics data, a multiblock PLS analysis followed by a multiblock sPLS could be conducted. Despite that the tools exist to build the model in R packages MixOmics and RGCCA, the tools currently required to accurately test for the goodness of fit of the PLS and to select the number of variables to keep in the multiblock sPLS are, unfortunately, not yet developed.

## Conclusions

Broilers reaching a higher BW at 35 days showed a lower inflammation and increased anti-oxidative defenses in jejunal tissue as determined by pathway analysis of RNAseq data. The microbiota in ileal and caecal digesta were poorly associated with BW, while the metabolome in these segments showed no association with BW. mRNA expression in caecal tonsil tissue showed a greater immune response in heavier broilers, potentially affecting or resulting from the microbiota. To improve broiler growth, reducing inflammation and increasing anti-oxidative defenses in the proximal small intestine should be further investigated.

## Funding

This study was funded by Idena (Sautron, France), and the “10.13039/501100003032Association Nationale Recherche Technologie” (10.13039/501100003032ANRT) through the Convention Industrielle de Formation par la RecherchE (CIFRE) grant no. 2021-0384. The funding bodies played no role in the design, analysis and reporting of the study.

Supplementary figures captions

**Supplementary Figure 1.** Association between body weight (BW) and ileal as well as caecal digesta microbiota α-diversity indexes in 35-day-old male Ross 308 broilers fed a non-starch polysaccharide-rich diet supplemented or not with either a probiotic or its derived postbiotic. (**A**) Table of p-values and BW effect size. (**B**) Plot of BW and caecal digesta microbiota α-diversity indexes showing an association with BW at *p* < 0.1.

## CRediT authorship contribution statement

**Samuel C.G. Jansseune:** Writing – review & editing, Writing – original draft, Visualization, Project administration, Methodology, Investigation, Funding acquisition, Formal analysis, Data curation, Conceptualization. **Wouter H. Hendriks:** Writing – review & editing, Supervision, Project administration, Methodology, Conceptualization.

## Disclosures

Although one of the authors (SCGJ) was a PhD candidate employed by Idena, the authors attest that they were completely free to independently design the study and collect, analyse and interpretate the data as well as write the manuscript.

## Data Availability

The datasets used and/or analyzed during the current study are available from the corresponding author on reasonable request.

## Appendix Supplementary materials

**Supplementary Figure 2**. Multidimensional scaling plot of ileal and caecal digesta microbiota β-diversity indexes in 35-day-old male Ross 308 broilers fed a non-starch polysaccharide-rich diet supplemented or not with either a probiotic or its derived postbiotic. Each dot represents an individual broiler. Image, image 1 dummy alt text

**Supplementary Figure 3.** Partial least square discriminant analysis Q2 criterion for the ileal (left) and caecal (right) digesta microbiota to explain body weight in 35-day-old male Ross 308 broilers fed a non-starch polysaccharide-rich diet supplemented or not with either a probiotic or its derived postbiotic. The horizontal black line is at 0.0975. Image, image 2 dummy alt text

**Supplementary Figure 4.** Partial least square discriminant analysis Q2 criterion for the semi-polar metabolome in ileum (left) and caecal (right) digesta to explain body weight in 35-day-old male Ross 308 broilers fed a non-starch polysaccharide-rich diet supplemented or not with either a probiotic or its derived postbiotic. The horizontal black line is at 0.0975. Image, image 3 dummy alt text

Image, image 4 dummy alt text

## Footnotes

Supplementary material associated with this article can be found, in the online version, at doi:10.1016/j.psj.2026.106463.
