# Supplementary figures and images for: Body weight of 35-day-old broilers is associated with proximal small intestinal inflammatory and oxidative pathways – A multi-omics study
Source: Poult Sci. 2026 Jan 18;105(4):106463. doi: 10.1016/j.psj.2026.106463 (PMC12865571; doi:10.1016/j.psj.2026.106463)

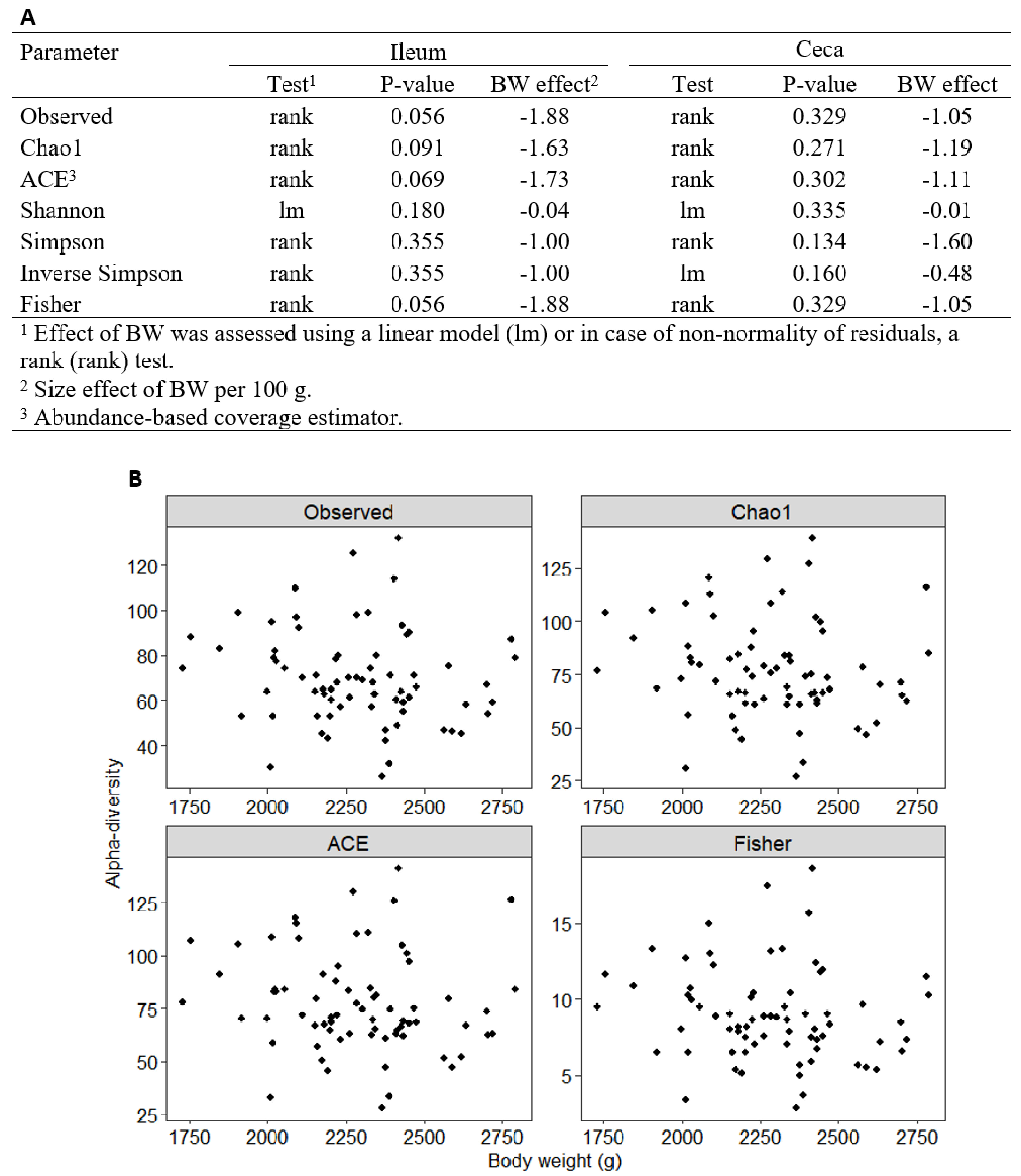

Supplement: Supplementary file 1 — Supplementary Figure 2. Multidimensional scaling plot of ileal and caecal digesta microbiota β-diversity indexes in 35-day-old male Ross 308 broilers fed a non-starch polysaccharide-rich diet supplemented or not with either a probiotic or its derived postbiotic. Each dot represents an individual broiler. [file mmc1.jpg]

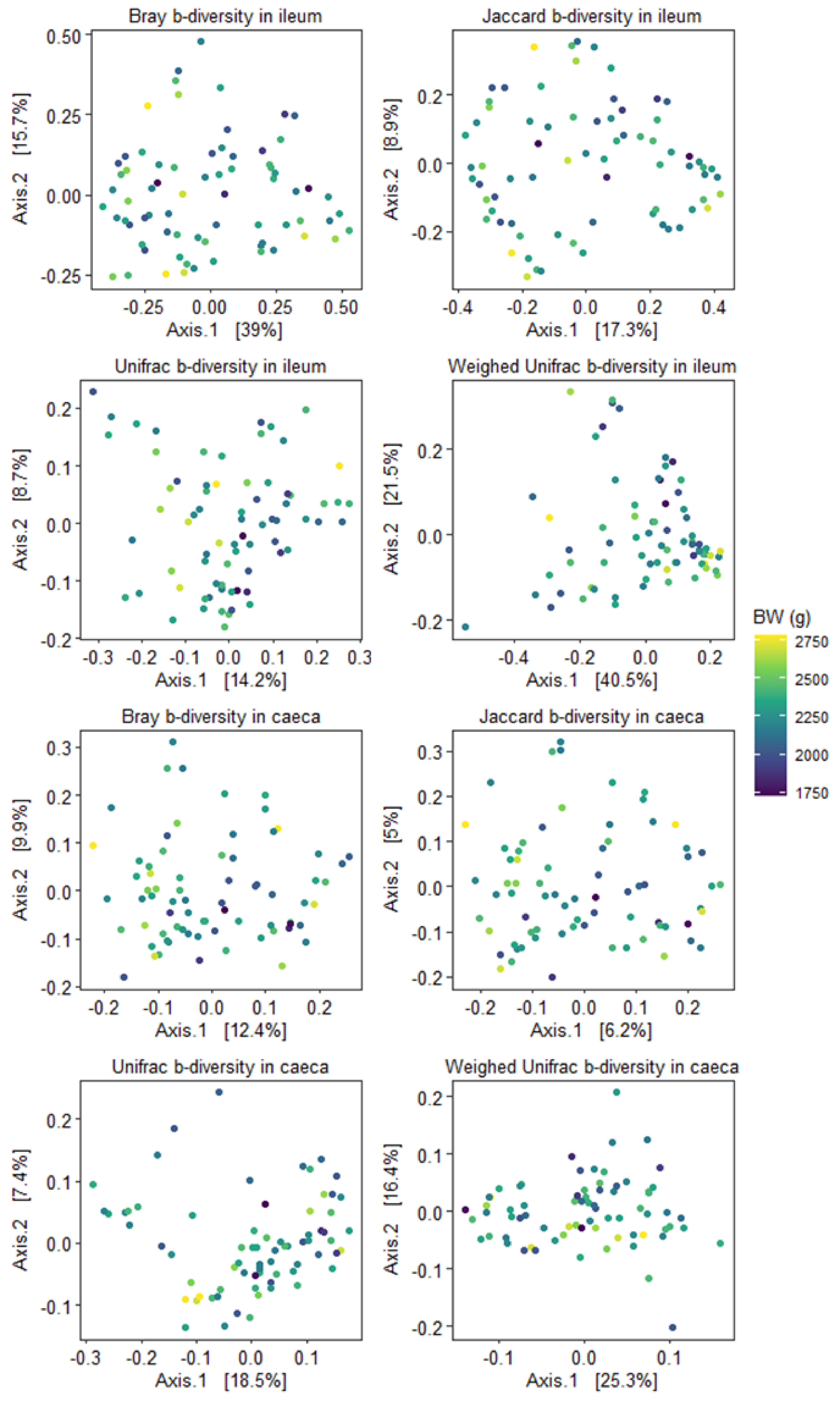

Supplement: Supplementary file 2 — Supplementary Figure 3. Partial least square discriminant analysis Q2 criterion for the ileal (left) and caecal (right) digesta microbiota to explain body weight in 35-day-old male Ross 308 broilers fed a non-starch polysaccharide-rich diet supplemented or not with either a probiotic or its derived postbiotic. The horizontal black line is at 0.0975. [file mmc2.jpg]

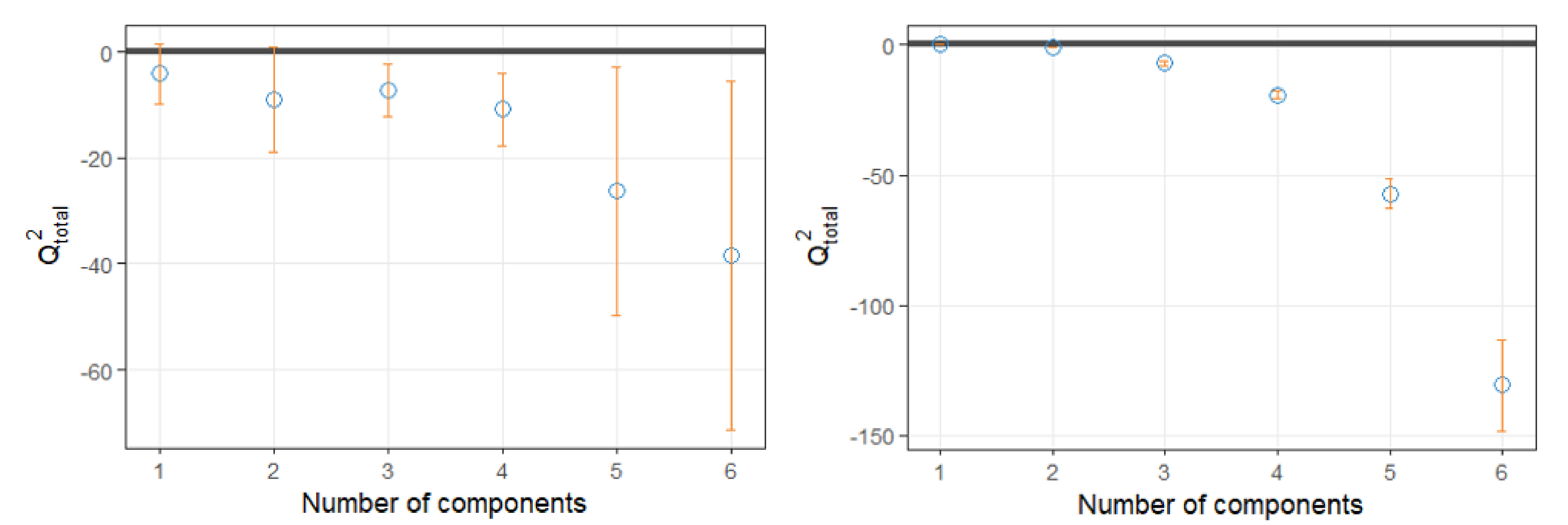

Supplement: Supplementary file 3 — Supplementary Figure 4. Partial least square discriminant analysis Q2 criterion for the semi-polar metabolome in ileum (left) and caecal (right) digesta to explain body weight in 35-day-old male Ross 308 broilers fed a non-starch polysaccharide-rich diet supplemented or not with either a probiotic or its derived postbiotic. The horizontal black line is at 0.0975. [file mmc3.jpg]

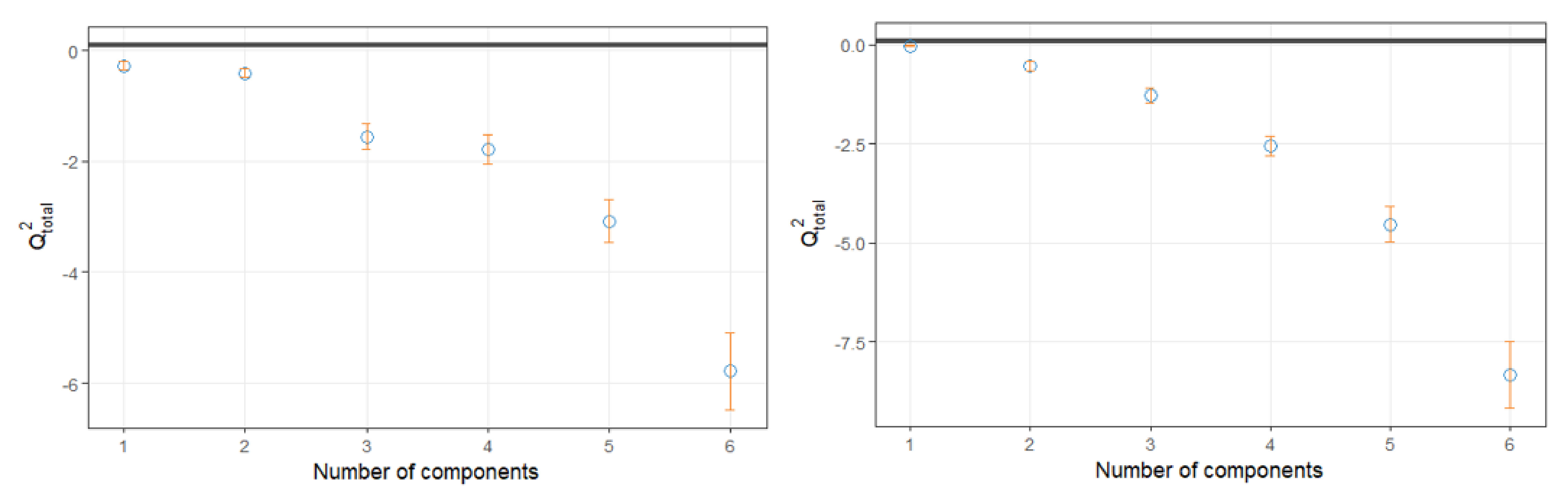

Supplement: Supplementary file 4 [file mmc4.jpg]
